# Role of Lipid Profile and Its Relative Ratios (Cholesterol/HDL-C, Triglyceride/HDL-C, LDL-C/HDL-C, WBC/HDL-C, and FBG/HDL-C) on Admission Predicts In-Hospital Mortality COVID-19

**DOI:** 10.1155/2023/6329873

**Published:** 2023-03-06

**Authors:** Jafar Mohammadshahi, Hassan Ghobadi, Golchin Matinfar, Mohammad Hossein Boskabady, Mohammad Reza Aslani

**Affiliations:** ^1^Department of Infectious Diseases and Tropical Medicine, School of Medicine, Ardabil University of Medical Sciences, Ardabil, Iran; ^2^Lung Diseases Research Center, Ardabil University of Medical Sciences, Ardabil, Iran; ^3^Faculty of Medicine, Ardabil University of Medical Sciences, Ardabil, Iran; ^4^Applied Biomedical Research Center, Mashhad University of Medical Sciences, Mashhad, Iran

## Abstract

**Background:**

Lipid profile and its related ratios such as total cholesterol (TC), low-density lipoprotein-cholesterol (LDL-C), triglyceride (TG), high-density lipoprotein-cholesterol (HDL-C), TG/HDL-C ratio, TC/HDL-C ratio, LDL-C/HDL-C ratio, white blood cell (WBC)/HDL-C ratio, and fasting blood glucose (FBG)/HDL-C ratio are valuable indicators that have been studied in various disorders to predict mortality. The present study was conducted with the aim of investigating the role of lipid profile ratios in predicting mortality in COVID-19 patients.

**Methods:**

At the beginning of hospitalization, laboratory tests were taken from all patients (*n* = 300). The ability of lipid profile ratios to determine the COVID-19 severity was evaluated using receiver-operating characteristic (ROC). In addition, survival probability was determined with the average of Kaplan-Meier curves, so that the end point was death.

**Results:**

In deceased patients, TG, TC, LDL-C, HDL-C, TC/HDL-C, TG/HDL-C, and LDL-C/HDL-C parameters were significantly lower than those of surviving patients, while WBC/HDL-C and FBG/HDL-C were significantly higher. TC (HR = 3.178, 95%CI = 1.064 to 9.491, *P* < 0.05), TG (HR = 3.276, 95%CI = 1.111 to 9.655, *P* < 0.05), LDL-C (HR = 3.207, 95%CI = 1.104 to 9.316, *P* < 0.05), and HDL-C (HR = 3.690, 95%CI = 1.290 to 10.554, *P* < 0.05), as well as TC/HDL-C (HR = 3.860, 95%CI = 1.289 to 11.558, *P* < 0.05), TG/HDL-C (HR = 3.860, 95%CI = 1.289 to 11.558, *P* < 0.05), LDL-C/HDL-C (HR = 3.915, 95%CI = 1.305 to 11.739, *P* < 0.05), WBC/HDL-C (HR = 3.232, 95%CI = 1.176 to 8.885, *P* < 0.05), and FBG/HDL-C ratios (HR = 4.474, 95%CI = 1.567 to 12.777, *P* < 0.01), were detectably related to survival. The multivariate Cox regression models showed that only FBG/HDL-C ratio (HR = 5.477, 95%CI = 1.488 to 20.153, *P* < 0.01) was significantly related to survival.

**Conclusion:**

The results suggested that FBG/HDL-C ratio in hospital-admitted COVID-19 patients was a reliable predictor of mortality.

## 1. Introduction

It has been revealed that mortality in patients with coronavirus disease 2019 (COVID-19) is related to increased inflammation and impaired immune response [1]. Many evidences showed that although most hospitalized patients with COVID-19 are mild, critically ill patients have a high mortality rate due to the development of multiorgan failure, acute respiratory disease, and septic shock [2]. Severity and morbidity in COVID-19 patients have been more evident in comorbidities such as cardiovascular disease, lung disease, diabetes, and kidney failure [3]. On the other hand, disturbance in lipid metabolism has been shown in the pathophysiology of many disorders such as cardiovascular, respiratory, and metabolic syndrome [4–8]. Defective lipid metabolism has also been reported in the COVID-19 [2]. Decreased levels of lipid profiles such as triglyceride (TG), total cholesterol (TC), high-density lipoprotein-cholesterol (HDL-C), and low-density lipoprotein-cholesterol (LDL-C) have been reported in COVID-19 patients, especially in severe patients [9, 10]. Dyslipidemia has also been evident in various system injuries such as cardiovascular, immune, and respiratory systems. In addition, high levels of proinflammatory cytokines are also important factors in developing dyslipidemia [11]. Due to the nature of COVID-19 (increased levels of proinflammatory cytokines and damage to various organs), lipid regulation is impaired.

In predicting the outcome of COVID-19, the use of various indicators has been investigated, such as systemic inflammation indexes [3]. On the other hand, lipid profile and related ratios such as TG/HDL-C, TC/HDL-C, and LDL-C/HDL-C, as well as white blood cell count (WBC)/HDL-C and first blood glucose (FBG)/HDL-C ratios in diagnosing severity and mortality of diseases, have been evaluated in various studies [12–14]. Therefore, the present study is aimed at investigating the predictive mortality in COVID-19 patients based on lipid profile ratios.

## 2. Method

This retrospective study was conducted in Ardabil Imam Khomeini Hospital, Ardabil, Iran, from July to September 2021. COVID-19 patients whose diagnosis was based on PCR test were included in the study. The study was conducted after it was approved by the Ethics Committee of Ardabil University of Medical Sciences (IR.ARUMS.MEDICINE.REC.1400.014).

The data collected included age, sex, medical history, laboratory tests, comorbidities, clinical symptoms, length of hospitalization, and disease outcome (recovery or death) of 300 COVID-19 patients by two trained medical students. The laboratory tests that were obtained from the patients in the first 24 hours, such as total white blood cell (WBC), neutrophil (NT), lymphocyte (LY), monocyte (MN), and platelet (Plt) counts, as well as hematocrit (Hct), hemoglobin (Hb), partial thromboplastin time (PTT), prothrombin time (PT), international normalized ratio (INR), fasting blood glucose (FBG), alkaline phosphatase (ALP), alanine transaminase (ALT), AST, erythrocyte sedimentation rate (ESR), D-dimer, lactate dehydrogenase (LDH), ferritin, urea, creatinine (Cr), sodium (Na), potassium (K), triglyceride (TG), low-density lipoprotein-cholesterol (LDL-C), total cholesterol (TC), and high-density lipoprotein-cholesterol (HDL-C), were analyzed.

In addition, lipid profile ratios were also calculated for all subjects, including TC/HDL-C ratio, TG/HDL-C ratio, LDL-c/HDL-C ratio, WBC/HDL-C ratio, NT/HDL-C ratio, LY/HDL-C ratio, MN/HDL-C ratio, and FBG/HDL-C ratio.

### 2.1. Data Analysis

For variables with normal distribution, mean ± standard deviation (SD) and, for variables abnormally distributed, median values and interquartile range (IQR) were used. Independent group *t*-test or Mann–Whitney test was used to compare variables. According to the Youden index, receiver-operating characteristic (ROC) curve analysis was conducted to estimate optimal values of cutoff, as well as to maximize sensitivity and specificity. Time zero in the current study was defined as the time of hospitalization for survival analysis. In order to avoid linear bias with univariate analysis for Charlson's index, lipid profile ratios were evaluated separately so that in case of *P* < 0.2, confounding factors were corrected. The probability of survival was estimated for the lipid profile ratios with the end point of death using the mean of the Kaplan-Meier curves. Finally, the Cox proportional hazards regression was used for both univariate and multivariate analysis. Data analysis was done by MedCalc version 19.4.1 and SPSS software.

## 3. Results

In the current study, 300 patients with COVID-19 were included, with an average age of 54.57 ± 17.02 (Table 1). The percentage of hospitalized men (58.7%) was higher than that of women (41.3%). The average hospital stay was 9.13 ± 4.21 days.

Laboratory findings of total WBC, Hb, Hct, Plt, PT, PTT, INR, Cr, Na, and K were in the normal range. However, ALT, AST, LDH, ferritin, ESR, urea, D-dimer, FBG, and ALP were higher than the normal range, while lymphocytes were less. The levels of lipid profiles measured at the beginning of hospitalization of the patients were TC: 128.37 ± 9.43, TG: 118.59 ± 15.12, LDL-C: 62.64 ± 4.82, and HDL-C: 42.50 ± 0.39 (Table 1).

### 3.1. Clinical Outcomes

**Figure 1:**
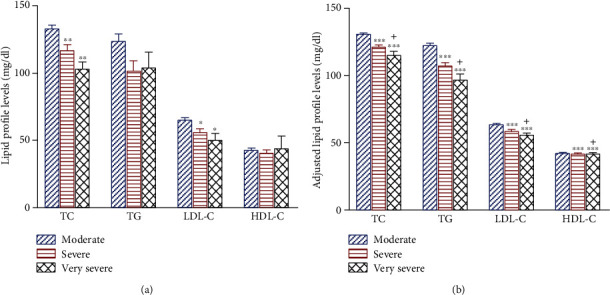
Mean + SEM of lipid profile levels of (a) baseline and (b) adjusted for the Charlson comorbidity index in moderate (blue), severe (red), and very severe COVID-19 patients (dark). TC: total cholesterol; TG: triglyceride; LDL-C: low-density lipoprotein-cholesterol; HDL-C: high-density lipoprotein-cholesterol. ^∗∗∗^*P* < 0.001, moderate vs. other group. ^+^*P* < 0.05, severe vs. very severe. For each group, *n* = 5.

The COVID-19 severity in 14 patients (4.7%) was very severe, in 61 patients (20.3%) severe, and in 225 patients (75%) moderate. Interestingly, it was revealed that the lipid profile (TG, TC, HDL-C, and LDL-C) was significantly lower in very severe and severe compared to moderate patients (both, *P* < 0.001) (Figure 1). Out of three hundred patients, 274 (91.3%) were discharged, and 26 died (8.7%).

### 3.2. Laboratory Finding Based on Outcome

The results showed that the following parameters were detectably higher in the dead than in the surviving patients: hospitalization stay (*P* < 0.001), age (*P* < 0.001), total WBC (*P* < 0.001), ALT (*P* < 0.05), AST (*P* < 0.05), ferritin (*P* < 0.05), FBG (*P* < 0.001), urea (*P* < 0.001), Cr (*P* < 0.05), and ALP (*P* < 0.01). On the other hand, the lymphocyte count in deceased patients was significantly lower than in surviving patients (*P* < 0.001).

The results of lipid profile and related ratios revealed that in patients who died compared to those who recovered, decreased levels occurred such as TC, TG, LDL-C, HDL-C, LDL-C/HDL-C ratio, TG/HDL-C ratio, and Lymphocyte/HDL-C ratio (for all *P* < 0.001) (Table 2). Interestingly, WBC/HDL-C and FBG/HDL-C ratios were significantly higher in deceased patients (both, *P* < 0.001). In addition, the analysis of the results revealed that the FBG/HDL-C ratio was not significantly different between male and female.

### 3.3. Receiver-Operating Characteristics (ROC)

**Figure 2:**
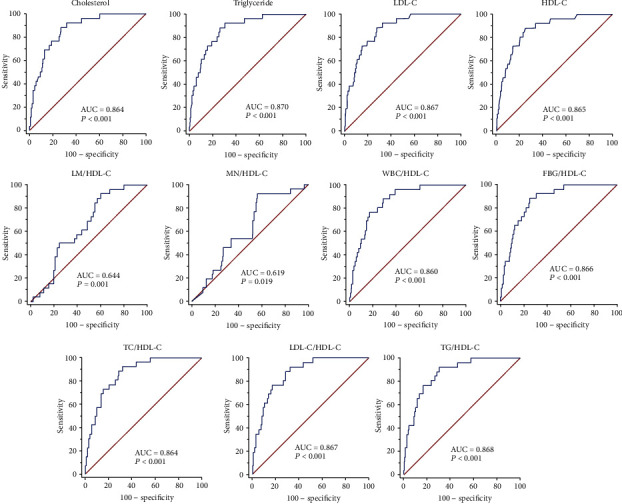
Receiver-operating characteristics curve for TC, TG, LDL-C, HDL-C, LM/HDL-C, MN/HDL-C, WBC/HDL-C, FBG/HDL-C, TC/HDL-C, LDL-C/HDL-C, and TG/HDL-C. FBG: fasting blood glucose; HDL-C: high-density lipoprotein-cholesterol; LDL-C: low-density lipoprotein-cholesterol; LM: lymphocyte; MN: monocyte; TC: total cholesterol; TG: triglyceride; WBC: white blood cell.

In the ROC-based analysis for survival assessment, the optimal cut-off values identified for lipid profile and its ratios were as follows: TC (≤123.97), TG (≤111.47), LDL-C (≤60.41), HDL-C (≤42.35), TG/HDL-C ratio (≤2.74), TC/HDL-C ratio (≤2.99), LDL-C/HDL-C ratio (≤1.45), WBC/HDL-C ratio (>169.64), lymphocyte/HDL-C ratio (≤27.88), monocyte/HDL-C ratio (≤4.72), and FBG/HDL-C ratio (>3.42) (Figure 2 and Table 3). In addition, AUD level was significant for TC (0.864), TG (0.870), LDL-C (0.867), HDL-C (0.865), TC/HDL-C ratio (0.864), TG/HDL-C ratio (0.868), LDL-C/HDL-C ratio (0.867), WBC/HDL-C ratio (0.860), lymphocyte/HDL-C (0.644), monocyte/HDL-C ratio (0.619), and FBG/HDL-C ratio (0.866) (Figure 2 and Table 3). The results revealed that AUC values were significantly higher for TC/HDL-C, TG/HDL-C, LDL-C/HDL-C, WBC/HDL-C, and FBG/HDL-C ratios than LM/HDL-C and MN/HDL-C ratios.

**Figure 3:**
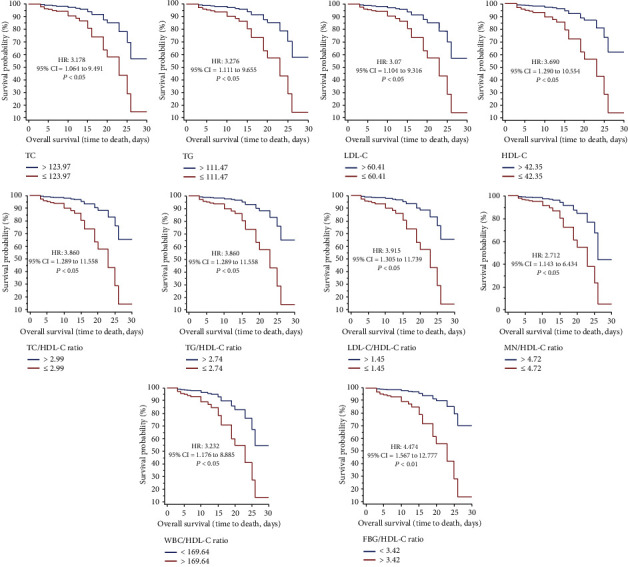
The Kaplan–Meier survival curves during hospitalization of COVID-19 patients with different cut-off values of the lipid profile ratios investigated. FBG: fasting blood glucose; HDL-C: high-density lipoprotein-cholesterol; LDL-C: low-density lipoprotein-cholesterol; LM: lymphocyte; MN: monocyte; TC: total cholesterol; TG: triglyceride; WBC: white blood cell.

The results of the Kaplan-Meier survival curves indicated that low survival was evident with low values of TC (HR = 3.178, 95%CI = 1.064 to 9.491, *P* < 0.05), TG (HR = 3.276, 95%CI = 1.111 to 9.655, *P* < 0.05), LDL-C (HR = 3.207, 95%CI = 1.104 to 9.316, *P* < 0.05), HDL-C (HR = 3.690, 95%CI = 1.290 to 10.554, *P* < 0.05), TC/HDL-C ratio (HR = 3.860, 95%CI = 1.289 to 11.558, *P* < 0.05), TG/HDL-C ratio (HR = 3.860, 95%CI = 1.289 to 11.558, *P* < 0.05), LDL-C/HDL-C ratio (HR = 3.915, 95%CI = 1.305 to 11.739, *P* < 0.05), WBC/HDL-C ratio (HR = 3.232, 95%CI = 1.176 to 8.885, *P* < 0.05), MN/HDL-C (HR = 2.712, 95%CI = 1.143 to 6.434, *P* < 0.05), and FBG/HDL-C ratio (HR = 4.474, 95%CI = 1.567 to 12.777, *P* < 0.01) (Figure 3 and Table 4). The multivariate Cox regression models showed that only FBG/HDL-C ratio (HR = 5.477, 95%CI = 1.488 to 20.153, *P* < 0.01) was significantly related to survival.

## 4. Discussion

In summary, the findings of the current study in COVID-19 patients were as follows:

1. In deceased patients, the values of TG, TC, LDL-C, and HDL-C levels, as well as TC/HDL-C, LDL-C/HDL-C, TG/HDL-C, LM/HDL-C, and MN/HDL-C ratios, were significantly less, but WBC/HDL-C and FBG/HDL-C ratios were higher than recovered subjects
2. The ROC and Kaplan-Meier survival curves identified that lipid profile (TC, TG, LDL-C, and HDL-C) and its related ratios (TC/HDL-C, TG/HDL-C, LDL-C/HDL-C, WBC/HDL-C, LM/HDL-C, MN/HDL-C, and FBG/HDL-C) were detectably related to survival
3. Only FBG/HDL-C ratio was significantly related to survival based on the multivariate Cox regression model

Various studies have shown that one of the characteristics of COVID-19 and its associated mortality is immune system dysfunction and severe inflammatory response [15, 16]. Systemic inflammation, sepsis, and metabolic disorders have been reported in severe COVID-19 [17]. Lipid metabolism disruption has been shown to affect the severity of COVID-19 [18]. As a result, it is believed that the disruption of the quantity and activity of the lipid profile may affect mortality due to COVID-19. Considerable evidence has shown that altered levels of lipid profiles, although contradictory, have been evident in COVID-19 patients [2, 12]. In most studies, reduced LDL-C, HDL-C, and TC levels have been reported in patients with COVID-19, especially in severe and critically ill patients [19]. The change in lipid profile levels in viral infections (such as dengue (DENV), *Helicobacter pylori* infection, sepsis, human immunodeficiency (HIV), nosocomial infections, and hepatitis B viruses (HBV)) has been considered an indicator of disease prognostics [9]. The prognostic role of various biomarkers in predicting the outcome of the disease has been discussed in COVID-19 patients [3].

In line with other studies, increased levels of leukocytes, AST, ALT, FBG, ALP, and urea were evident in deceased patients compared to recovered patients [3]. Interestingly, reduced TG, TC, LDL-C, and HDL-C levels were also observed in deceased patients. Compared with mild COVID-19, reduced levels of lipid profiles have been shown in the severe and mortality groups [20–22]. In COVID-19 patients hospitalized in ICU, it has also been demonstrated that reduced levels of HDL were associated with high mortality [23].

Cholesterol plays an important role in SARS-CoV-2 infection through interaction with SARS-CoV-19 S protein [24]. In addition, it has been found that HDL-C exerts an important role in host defense against viral, parasitic, and bacterial infections through anti-inflammatory effects [9]. The anti-inflammatory effects of HDL-C have been revealed by reducing the activity of T-cells and the expression level of inflammatory factors in macrophages, inhibiting the activation of monocytes and the expression of adhesion molecules (such as vascular cell adhesion molecule 1 (VCAM-1), intercellular adhesion molecule 1 (ICAM-1), E-selectin, and P-selectin) [25–27]. In addition, other effects observed in relation to HDL-C are as follows: antiapoptotic, antioxidative, antiviral, and antithrombotic effects [9]. However, the HDL-C beneficial effects can be impaired in inflammatory conditions [28]. Proinflammatory cytokines such as CRP and IL-6 can reduce HDL-C production by inhibiting apolipoprotein synthesis enzyme activity [29]. It seems that in SARS-CoV-2 infection, lipid profile changes affect the severity of the disease, which requires additional investigations.

The current study also decreased levels of LDL-C, TC, and TG. Low TG, TC, and LDL-C levels are observed which could be considered malnutrition markers [30]. In addition, it has also been shown that inflammation leads to lower LDL-C levels [2]. In the current study, hypocholesterolemia appears to be a result of malnutrition or the cytokine storm caused by SARS-CoV-2 infection.

Recently, lipid profile ratios have been used in various studies as predictors of the outcome of diseases [12, 31]. Decreased TG/HDL-C, TC/HDL-C, LDL-C/HDL-C, LM/HDL-C, and MN/HDL-C ratios and increased WBC/HDL-C and FBG/HDL-C ratios were markedly observed in the mortality group. In addition, it was identified that survival was related to TC, TG, HDL-C, and LDL-C levels, as well as TC/HDL-C, TG/HDL-C, LDL-C/HDL-C, LM/HDL-C, MN/HDL-C, WBC/HDL-C, and FBG/HDL-C ratios. On the other hand, LM/HDL-C and MN/HDL-C ratios were the lowest in predicting disease severity. Multivariate Cox regression analysis identified that only FBG/HDL-C ratio remained significant with survival. Much evidence has reported that comorbidities such as cardiovascular, diabetes, and kidney diseases have affected the severity of COVID-19 [3]. Hyperglycemia has been shown to exacerbate inflammatory conditions through increased inflammatory markers, oxidative stress, clotting factors, and LDL glycation [12, 32, 33]. It can be concluded that the increase in FBG and the decrease in HDL-C levels in patients with COVID-19 were more significant in predicting mortality than other ratios of lipid profile.

The limitations of the study were as follows: (1) selection of patients from one center, (2) the data were collected from the electronic record system, (3) difference in the severity of the disease during hospitalization, (4) the possibility of the effects of different variants of the results, and (5) sample size limitation.

## 5. Conclusions

In conclusion, the results showed that lipid profile ratios were valuable in diagnosing COVID-19 severity. The FBG/HDL-C ratio among lipid profile ratios had a higher power of predicting COVID-19 mortality. It seems that the use of lipid profile ratios and changes in glucose levels in SARS-CoV-2 infection is more important than other changes in predicting the mortality of patients.

## Figures and Tables

**Table 1:** Demographic, hematological, lipid profile and its relative ratios of COVID-19.

Variables	Normal range	COVID-19 (*n* = 300)
Age	—	54.57 ± 17.02
Sex	—	
Male, *N* (%)		176 (58.7)
Female, *N* (%)		124 (41.3)
Hospitalization stay	—	9.13 ± 4.21
WBC (×10^9^/L)	3.5-9.5	6.20 (4.35-8.55)
Adjusted WBC	3.5-9.5	6.67 (6.39-7.13)
Neutrophil (×10^9^/L)	1.8-6.3	5.54 (5.00-5.89)
Lymphocyte (×10^9^/L)	1.1-3.2	0.94 (0.67-1.40)
Monocyte (×10^9^/L)	0.2-0.3	0.14 (0.13-0.27)
Hb (mg/mL)	11.5-15	13.00 (11.5-14.0)
Hct (%)	36-48	37 (33.0-41.0)
PLT (×10^9^/L)	125-350	178 (140-245)
PT (s)	11-13.5	12.0 (12-13.0)
PTT	30-40	30 (30-32)
INR	0.8-1.1	1 (1-1.0)
ALT (IU/L)	7-40	42 (26-72)
AST (IU/L)	0-45	52 (30-71.5)
LDH (IU/L)	114-240	650 (477-785)
Ferritin (*μ*g/L)	11-330	890 (654-998)
ESR (mm/hr)	0-29	51 (38-65)
FBG (mg/mL)	70-100	110 (96-140)
Urea (mg/mL)	6-24	35 (25-51)
Cr (mg/mL)	0.5-1.2	1.0 (0.90-1.20)
D.dimer (mg/L)	0-0.5	0.75 (0.5-1.5)
ALP (IU/L)	44-147	179 (150-245)
Na (mEq/L)	135-145	139 (136-142)
K (mEq/L)	3.5-5.3	3.9 (3.7-4.0)
TC (mg/dL)	Less than 200	128.37 ± 9.43
TG (mg/dL)	10-150	118.59 ± 15.12
LDL-C (mg/dL)	70-130	62.64 ± 4.82
HDL-C (mg/dL)	40-60	42.50 ± 0.39
TC/HDL-C	—	3.02 ± 0.19
TG/HDL-C	—	2.79 ± 0.33
LDL-C/HDL-C	—	1.47 ± 0.10
WBC/HDL-C	—	159.83 ± 11.83
NT/HDL-C	—	127.13 ± 15.37
LM/HDL-C	—	25.97 ± 14.47
MN/HDL-C	—	4.30 ± 2.85
FBG/HDL-C	—	3.15 ± 0.57
Severity	—	
Moderate, *N* (%)		225 (75)
Severe, *N* (%)		61 (20.3)
Very severe, *N* (%)		14 (4.7)
Comorbidities	—	
Cardiovascular disease (%)		22.7
Respiratory disease (%)		14.7
Kidney disease (%)		8.3
Diabetes (%)		20.4
Cancer (%)		5.0
Liver (%)		3.0
Charlson's comorbidity index		2 (0.00-5.00)
Outcome	—	
Survival, *N* (%)		274 (91.3)
Death, *N* (%)		26 (8.7)

ALP: alkaline phosphatase; ALT: alanine transaminase; AST: aspartate transaminase, Cr: creatinine; ESR: erythrocyte sedimentation rate; FBG: fasting blood glucose; Hb: hemoglobin; Hct: hematocrit; HDL-C: high-density lipoprotein-cholesterol; INR: international normalized ratio; LDH: lactate dehydrogenase; LDL-C: low-density lipoprotein-cholesterol; LM: lymphocyte; MN: monocyte; PLT: platelet; PT: prothrombin time; PTT: partial thromboplastin time; TC: total cholesterol; TG: triglyceride; WBC: white blood cell.

**Table 2:** Demographic, hematological, lipid profile and its relative ratios of COVID-19 in survivor and nonsurvivor patients.

Variables	COVID-19		*P* value
	Survival (*N* = 274)	Death (*N* = 26)	
Age	52.93 ± 16.48	71.92 ± 12.52	≤0.001
Sex			0.837
Male, *N* (%)	160 (90.0)	16 (9.1)	
Female, *N* (%)	114 (91.9)	10 (8.1)	
Hospitalization stay	8.78 ± 3.50	12.88 ± 7.85	≤0.001
WBC (×10^9^/L)	6.73 ± 4.09	7.34 ± 3.79	≤0.001
Neutrophil (×10^9^/L)	5.39 ± 0.07	5.45 ± 0.03	0.562
Lymphocyte (×10^9^/L)	1.13 ± 0.06	0.81 ± 0.02	≤0.001
Monocyte (×10^9^/L)	0.18 ± 0.01	0.15 ± 0.01	0.172
Hb (mg/mL)	12.82 ± 1.95	12.14 ± 2.13	0.092
Hct (%)	37.10 ± 5.43	35.34 ± 6.00	0.120
PLT (×10^9^/L)	197.64 ± 83.47	175.62 ± 65.41	0.192
PT (s)	12.56 ± 0.83	12.76 ± 0.88	0.256
PTT	31.46 ± 3.45	31.07 ± 1.89	0.570
INR	1.03 ± 0.11	1.05 ± 0.12	0.510
ALT (IU/L)	57.22 ± 57.54	76.46 ± 36.88	≤0.05
AST (IU/L)	60.58 ± 54.68	77.50 ± 32.23	≤0.05
LDH (IU/L)	665.54 ± 235.68	697.65 ± 174.62	0.499
Ferritin (*μ*g/L)	855.60 ± 295.26	980.58 ± 302.19	≤0.05
ESR (mm/hr)	53.18 ± 39.10	55.38 ± 17.18	0.777
FBG (mg/mL)	130.98 ± 20.92	164.71 ± 20.43	≤0.001
Urea (mg/mL)	41.12 ± 29.07	70.26 ± 41.05	≤0.001
Cr (mg/mL)	1.27 ± 1.27	1.80 ± 1.40	≤0.05
D.dimer (mg/L)	977.69 ± 737.39	1271.7 ± 805.62	0.055
ALP (IU/L)	201.59 ± 83.02	254.96 ± 85.28	≤0.01
Na (mEq/L)	138.89 ± 3.66	139.73 ± 5.13	0.284
K (mEq/L)	3.93 ± 0.44	4.18 ± 0.63	0.065
TC (mg/mL)	130.30 ± 37.89	108.15 ± 20.76	≤0.001
TC adjusted	129.58 ± 8.60	115.63 ± 8.43	≤0.001
TG (mg/mL)	120.11 ± 70.80	102.77 ± 36.14	≤0.05
TG adjusted (mg/mL)	120.55 ± 13.73	97.92 ± 13.73	≤0.001
LDL-C (mg/mL)	63.27 ± 22.72	56.00 ± 20.98	0.104
LDL-C adjusted (mg/mL)	63.26 ± 4.39	56.08 ± 4.30	≤0.001
HDL-C (mg/mL)	42.47 ± 14.95	42.84 ± 9.96	0.902
HDL-C adjusted (mg/mL)	42.55 ± 0.35	41.98 ± 0.39	≤0.001
TC/HDL-C	3.04 ± 0.17	2.75 ± 0.17	≤0.001
TG/HDL-C	2.83 ± 0.30	2.33 ± 0.30	≤0.001
LDL-C/HDL-C	1.48 ± 0.09	1.33 ± 0.09	≤0.001
WBC/HDL-C	158.38 ± 10.90	175.09 ± 10.55	≤0.001
NT/HDL-C	126.87 ± 15.84	129.80 ± 8.86	0.148
LM/HDL-C	26.60 ± 14.85	19.38 ± 6.97	≤0.001
MN/HDL-C	4.37 ± 2.90	3.62 ± 2.23	0.205
FBG/HDL-C	3.08 ± 0.52	3.92 ± 0.52	≤0.001
Severity			≤0.001
Moderate, *N* (%)	224 (99.6)	1 (0.4)	
Severe, *N* (%)	49 (80.3)	12 (19.7)	
Very severe, *N* (%)	1 (7.1)	13 (92.9)	
Comorbidities			
Cardiovascular disease (%)	18.6	65.4	≤0.001
Respiratory disease (%)	13.1	30.8	≤0.05
Kidney disease (%)	6.9	23.1	≤0.05
Diabetes (%)	22.6	57.7	≤0.001
Cancer (%)	4.0	15.4	≤0.05
Liver (%)	2.9	3.8	0.563

Abbreviations are similar to those of Table 1.

**Table 3:** Receiver-operating characteristics (ROC) curves and prognostic accuracy of lipid profile and its relative ratios in COVID-19.

Variables	AUC	95% CI	*P* value	Cut-off	Sensitivity	Specificity (%)
TC	0.864	0.820 to 0.901	<0.001	≤123.97	88.5	73
TG	0.870	0.826 to 0.906	<0.001	≤111.47	88.5	74.1
LDL-C	0.867	0.823 to 0.903	<0.001	≤60.41	88.5	72.6
HDL-C	0.865	0.822 to 0.902	<0.001	≤42.35	88.5	75.2
TC/HDL-C	0.864	0.820 to 0.900	<0.001	≤2.99	92.3	68.2
TG/HDL-C	0.868	0.825 to 0.905	<0.001	≤2.74	92.3	69.7
LDL/HDL-C	0.867	0.823 to 0.903	<0.001	≤1.45	92.3	67.9
WBC/HDL-C	0.860	0.815 to 0.897	<0.001	>169.64	76.9	83.2
NT/HDL-C	0.522	0.464 to 0.580	0.613	>124.18	84.6	39.1
LM/HDL-C	0.644	0.587 to 0.699	<0.001	≤27.88	92.3	40.1
MN/HDL-C	0.619	0.560 to 0.676	<0.05	≤4.72	92.3	44.1
FBG/HDL-C	0.866	0.822 to 0.902	<0.001	>3.42	88.46	75.18

AUC: area under curve; CI: confidence interval; FBG: fasting blood glucose; HDL-C: high-density lipoprotein-cholesterol; LDL-C: low-density lipoprotein-cholesterol; LM: lymphocyte; MN: monocyte; TC: total cholesterol; TG: triglyceride; WBC: white blood cell.

**Table 4:** Hazard ratios of the ratios under investigation obtained by Cox regression analysis in COVID-19 patients.

Variables	HR	95% CI	*P* value
TC	3.178	1.064 to 9.491	<0.05
TG	3.276	1.111 to 9.655	<0.05
LDL-C	3.207	1.104 to 9.316	<0.05
HDL-C	3.690	1.290 to 10.554	<0.05
TC/HDL-C	3.860	1.289 to 11.558	<0.05
TG/HDL-C	3.860	1.289 to 11.558	<0.05
LDL-C/HDL-C	3.915	1.305 to 11.739	<0.05
WBC/HDL-C	3.232	1.176 to 8.885	<0.05
LM/HDL-C	2.023	0.732 to 5.592	0.174
MN/HDL-C	2.712	1.143 to 6.434	<0.05
FBG/HDL-C	4.474	1.567 to 12.777	<0.01

HR: hazard ratio; FBG: fasting blood glucose; HDL-C: high-density lipoprotein-cholesterol; LDL-C: low-density lipoprotein-cholesterol; LM: lymphocyte; MN: monocyte; TC: total cholesterol; TG: triglyceride; WBC: white blood cell.

## Data Availability

The datasets used and/or analyzed during the current study are available upon reasonable request from the corresponding author.
